# Brown Adipose Tissue Prevalence Is Lower in Obesity but Its Metabolic Activity Is Intact

**DOI:** 10.3389/fendo.2022.858417

**Published:** 2022-03-31

**Authors:** Oana C. Kulterer, Carsten T. Herz, Marlene Prager, Christoph Schmöltzer, Felix B. Langer, Gerhard Prager, Rodrig Marculescu, Alexandra Kautzky-Willer, Marcus Hacker, Alexander R. Haug, Florian W. Kiefer

**Affiliations:** ^1^ Division of Nuclear Medicine, Department of Biomedical Imaging and Image-Guided Therapy, Medical University of Vienna, Vienna, Austria; ^2^ Division of Nephrology and Dialysis, Department of Medicine III, Medical University of Vienna, Vienna, Austria; ^3^ Division of Endocrinology and Metabolism, Department of Medicine III, Medical University of Vienna, Vienna, Austria; ^4^ Division of Visceral Surgery, Department of Surgery, Medical University of Vienna, Vienna, Austria; ^5^ Division of Medical-Chemical Laboratory Diagnostics, Department of Laboratory Medicine, Medical University of Vienna, Vienna, Austria

**Keywords:** brown adipose tissue, cold exposure, thermogenesis, obesity, PET/CT

## Abstract

Due to its high metabolic activity, brown adipose tissue (BAT) has become a promising target for the development of novel treatment concepts for metabolic disease. Despite several reports of a negative association between the presence of active BAT and obesity, very little is known about the quantitative and qualitative differences of BAT in lean and obese individuals. Systematic studies directly comparing cold-induced BAT activity in leanness and obesity are currently lacking. Here we studied BAT mass and function in 31 lean and 64 obese men and women. After a standardized cooling protocol using a water-perfused vest, ^18^F-FDG-positron emission tomography/computed tomography scans were performed, and BAT was delineated using lean body-mass adjusted standardized uptake value (SUV) thresholds in anatomic regions with fat radiodensity. Cold-induced thermogenesis (CIT), a functional readout of BAT activity, was quantified by indirect calorimetry. Active BAT was present in a significantly higher proportion of lean than obese individuals (58% vs. 33%, p=0.019). In these participants with active BAT, however, BAT volume and activity did not differ between leanness and obesity. Accordingly, CIT was similar in both weight groups. BAT metrics were not related to adiposity or total fat mass per se. However, in obese participants a strong negative correlation existed between visceral adipose tissue and BAT volume, ^18^F-FDG uptake and CIT. In summary, despite a significantly lower prevalence of BAT, the metabolic activity and thermogenic capacity of BAT appears to be still intact in obesity and is inversely associated with visceral fat mass.

## Introduction

Obesity occurs as a chronic imbalance between energy intake and energy expenditure. Whereas white adipose tissue (WAT) stores energy in form of triglycerides, brown adipose tissue (BAT) combusts excessive energy *via* uncoupled mitochondrial respiration, suggesting promotion of BAT activity as a promising target in the treatment of obesity and type 2 diabetes mellitus (T2DM) (1–4). The current gold standard for the detection and quantification of BAT in clinical studies is ^18^F-fluorodeoxyglucose (^18^F-FDG) positron emission tomography/computed tomography (PET/CT) (5). Based on the uptake of the glucose analog ^18^F-FDG in deep cervical, supraclavicular, axillary, mediastinal and paraspinal fat depots in participants undergoing standardized cooling protocols, we gathered a large body of knowledge about the prevalence, plasticity and potential metabolic benefits of human BAT during the last decade (6). The prevalence of active BAT decreases with age, the degree of adiposity as well as with impaired glucose and lipid metabolism (7, 8). BAT activity depends on seasonal variation and it can be recruited by chronic intermittent cooling protocols with potential benefits on body fat distribution and nutrient handling (9–13). The expansion of active BAT particularly in individuals with obesity might harbor the potential to counteract metabolic deterioration usually seen in this population, given the positive impact of BAT on energy homeostasis and beneficial BAT-derived hormones acting on peripheral insulin sensitivity and hepatic lipid handling (2, 14, 15). Whereas single studies have reported a negative association between obesity and the frequency of active BAT, systematic data on the prevalence and metabolic activity of BAT comparing lean and obese individuals are lacking. Particularly, the previous assumption that BAT is dysfunctional in obesity has recently been challenged (8, 16). In general, between-study comparability is poor due to different thresholding strategies and different regions of interest in addition to different cooling protocols. With the introduction of the BARCIST criteria the methodological bias of body fatness for the quantification of metabolic activity seen in previous studies using fixed SUV thresholds was reduced (5). In this study we addressed an unmet issue in BAT research, by investigating differences in BAT prevalence, metabolic activity and cold-induced thermogenesis in leanness and obesity using a well-characterized cohort of otherwise healthy individuals with a wide weight distribution undergoing personalized cooling protocols for BAT activation.

## Materials and Methods

### Study Approval and Procedures

For this study 95 lean and obese (otherwise healthy) volunteers were recruited through printed advertisement. Interested study volunteers were screened for medical history and underwent physical examination and those that met the inclusion criteria were enrolled in the study after providing written informed consent. Inclusion criteria were a BMI between 18.5 and 55 kg/m^2^ and an age between 20 and 50 years. Exclusion criteria included endocrinological disease except for substituted hypothyroidism, chronic kidney disease, chronic liver disease, chronic inflammatory conditions requiring systemic therapies, as well as the use of medications modifying adrenergic receptor signaling. This study has been approved by the Ethics Committee of the Medical University of Vienna (no. 1032/2013 and no. 1071/2017). The study was conducted at the Division of Endocrinology and Metabolism, Department of Medicine III, Medical University of Vienna in accordance with the principles of the Declaration of Helsinki.

After an overnight fast (> 10 hours), the volunteers arrived at the institution’s metabolic unit. All study participants were asked to refrain from physical exercise for 2 days before the study visit. After their arrival at the metabolic unit anthropomorphic measurements were performed, and an intravenous cannula was inserted in a peripheral vein for blood samples collection. Body composition was assessed using bioelectrical impedance analysis (seca mBCA 515, seca GmbH & Co. KG, Hamburg, Germany). After resting 30 minutes in supine position, resting energy expenditure (REE) was measured by indirect calorimetry (Quark RMR, COSMED srl., Rome, Italy). Then, the patients were fitted with a water-perfused cooling vest which covered the whole torso (CoolShirt Systems, Stockbridge, Georgia, USA). All participants were wearing 100% cotton t-shirts under the cooling vest. The temperature was gradually decreased until shivering was detected by electromyography (EMG Quattro, OT Bioeletronica, Torino, Italy) or the participant reported shivering and severe thermal discomfort, respectively. The EMG electrodes were placed on the major pectoral muscle. Shivering was assessed in real-time always by the same study personnel and identified by sudden increases in the amplitude of the EMG signal either continuously or in bursts which were not attributable to voluntary muscle contractions. At any signs of shivering the temperature of the cooling vest was increased by 1.12-2.24°C and we re-evaluated whether further temperature changes were necessary within 5-10 minutes. After 60 minutes, a second indirect calorimetry was performed to assess cold-induced thermogenesis (CIT), the percentage increase in resting energy expenditure after cold exposure compared to baseline. After the second indirect calorimetry volunteers received 2.5 Megabecquerel per kilogram of bodyweight of ^18^F-fluorodeoxyglucose (^18^F-FDG) intravenously followed by another 60 minutes of cold exposure during the tracer uptake. Then, a combined PET/CT acquisition was started as previously described (8, 17). The study participants underwent a total of 180 minutes cold exposure. All laboratory analyses were performed using routine assays at the institution’s department of laboratory medicine.

### Image Analysis

The PET/CT images were analyzed as previously described (8). Briefly, the regions of interest were delineated in the axial fusion images using a semi-automated segmentation protocol in accordance with the BARCIST criteria (5). Only regions of interest located within a CT radio density of –190 to –10 Hounsfield units (HU) and with a minimal standardized uptake value (SUV) higher than a personalized threshold of 1.2 divided by relative lean body mass were classified as active BAT. The resulting individualized SUV thresholds were in the range from 1.34 to 2.77. SUVmean values presented in this study were normalized to lean body mass as suggested in the BARCIST criteria (5). Each slice was visually inspected by two experienced nuclear medicine physicians to exclude spillover from adjacent non-fat tissues prone to ^18^F-FDG uptake such as muscles and glands. Abdominal adipose tissue was delineated by placing a volume of interest within fat radiodensity (-300 to – 10 HU) on the low dose CT along the level of the third lumbar vertebrae. The subcutaneous adipose tissue compartment was manually delineated as fat located external to the abdominal and back muscles. Visceral adipose tissue was obtained by subtracting the amount of subcutaneous adipose tissue from the entire fat depot.

### Statistics

Continuous data are presented as mean ± standard deviation or median ± interquartile range (IQR), as appropriate. Differences between two groups were tested using Student’s t-test, Mann-Whitney U test, or χ^2^ test. To investigate associations between two continuous variables, Spearman’s rank correlation coefficient was used. All analyses were performed using SPSS 25 (IBM Corp., Armonk, NY, USA) and GraphPad Prism 6.0 (GraphPad Software Inc., La Jolla, CA, USA). Two-sided p-values < 0.05 were deemed statistically significant.

## Results

### Lean Individuals Have a Higher Prevalence of Active BAT but Similar BAT Volume and Activity as Individuals With Obesity

In order to study the distribution of BAT in leanness and obesity, 95 volunteers (31 lean and 64 obese) underwent ^18^F-FDG-PET/CT scans after an individualized cooling protocol. Age and sex were evenly distributed between the two groups. Body mass index (BMI), body fat content, and metabolic laboratory parameters such as blood lipids, glucose homeostasis and c-reactive protein were significantly higher in the obesity group (**Table 1**).

**Table 1:** Baseline characteristics of the study cohort.

	Lean (n = 31)	Obese (n = 64)	P-value
**Age (years)**	29 ± 5	31 ± 9	0.152
**Sex (female)**	18 (58.1%)	38 (59.4%)	0.857
**BMI (kg/m^2^)**	22.3 ± 1.9	39.6 ± 6.6	<0.001
**WHR**	0.77 ± 0.07	0.95 ± 0.1	<0.001
**BF (%)**	23 ± 7	45 ± 8	<0.001
**Triglycerides (mg/dL)**	73 (52 – 116)	99 (76 - 132)	0.008
**Cholesterol (mg/dL)**	158 ± 28	176 ± 34	0.011
**HDL cholesterol (mg/dL)**	62 ± 17	46 ± 13	<0.001
**Glucose (mg/dL)**	86 ± 7	89 ± 8	0.041
**HOMA-IR**	1.10 (0.71 - 1.75)	3.39 (2.53 - 4.90)	<0.001
**CRP (mg/dL)**	0.11 (0.03 - 0.18)	0.30 (0.15 - 0.84)	<0.001
**TSH (µU/mL)**	2.08 (1.32 - 2.95)	1.83 (1.26 - 2.34)	0.222

BF, body fat; BMI, body mass index; CRP, c-reactive protein; HOMA-IR, homeostasis model assessment insulin resistance; HDL, high-density lipoprotein; TSH, thyroid stimulating hormone; WHR, waist-to-hip ratio. Data are depicted as count and column percent, mean ± standard deviation, or median (25th percentile-75th percentile). Between group differences were tested using χ^2^ -test, Student’s t-test, or Mann-Whitney-U-test, as appropriate.

**Figure 1:**
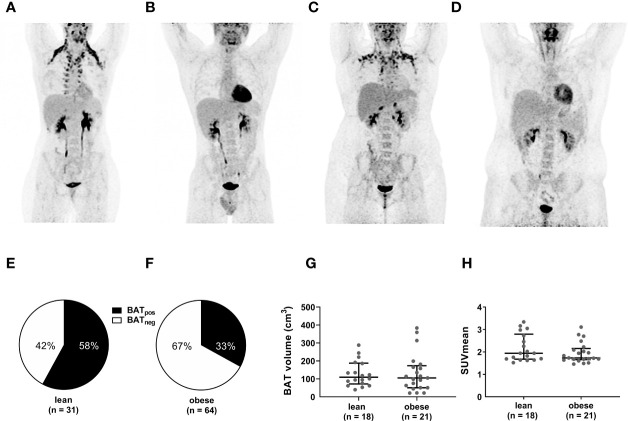
Lower frequency of active BAT but similar BAT volume and activity in obese versus individuals. Representative PET images of lean BAT_pos_ **(A)** and BAT_neg_ **(B)** as well as obese BAT_pos_ **(C)** and BAT_neg_ **(D)** participants. Compared to BAT_neg_ individuals, BAT_pos_ individuals exhibit significant ^18^F-FDG uptake in the deep cervical, supraclavicular, axillar, and paravetrebral fat depots. The pie charts depict the proportion of BAT_pos_ to BAT_neg_ participants in the lean **(E)** and the obese **(F)** cohort. BAT volume **(G)** and SUVmean **(H)** in lean and obese individuals with active BAT. The graphs indicate median values and interquartile ranges.

**Figure 2:**
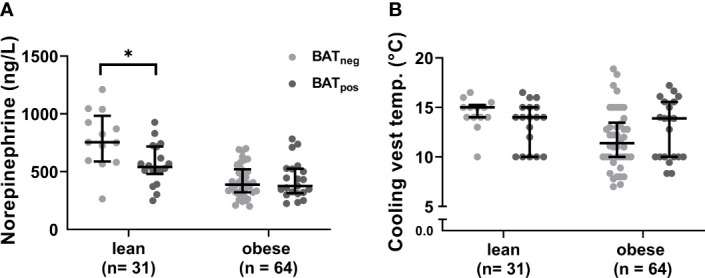
Cold-induced norepinephrine concentrations and cooling vest temperatures. Norepinephrine plasma levels after cold exposure in BAT_neg_ and BAT_pos_ participants within each weight group **(A)**. Cooling vests temperatures between lean and obese individuals **(B)**. The graphs indicate median values and interquartile ranges. *p ≤ 0.050.

After cold exposure, active BAT was detected in 58% of the lean participants but only 33% of the individuals with obesity (p=0.019). Active BAT as evidenced by ^18^F-FDG uptake was predominantly located in supraclavicular, cervical, axillary, and thoracic paraspinal fat depots in both groups (**Figures 1A–F**). Those with active BAT were termed BAT positive (BAT_pos_), those without any detectable BAT depots BAT negative (BAT_neg_). To exclude that insufficient cold-activation contributed to these differences, we analyzed circulating cold-induced norepinephrine concentrations. Norepinephrine concentrations were higher in BAT_neg_ versus BAT_pos_ lean participants and similar in both obese groups (**Figure 2A**). The temperatures of the water-perfused cooling vests did not differ between any of the study groups (**Figure 2B**). In summary, these data reassure that insufficient cooling was not the reason for the lack of FDG-uptake in BAT_neg_ individuals.

Next, we analyzed in BAT_pos_ participants whether PET-derived metrics of BAT activity differed between the lean and obese group. Both, the median BAT volume (110 (IQR: 75-186) vs. 105 (51- 165) p=0.662) and median SUVmean (1.94 (1.69-2.73) vs. 1.73 (1.68-2.09), p=0.284) were evenly distributed between lean and obese individuals (**Figures 1G, H**). Taken together, even though the prevalence of active BAT is lower in individuals with obesity, BAT volume and cold-mediated glucose uptake do not differ between lean and obese subjects.

### Cold-Induced Thermogenesis Is Similar in Lean and Obese Individuals

**Figure 3:**
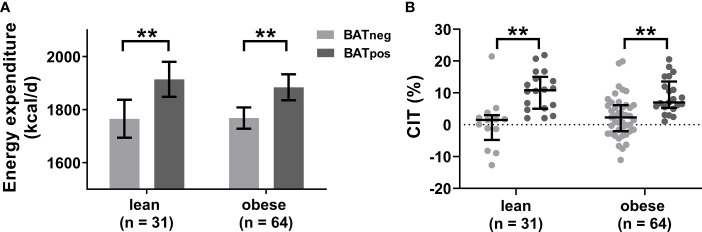
Cold-induced energy expenditure is similar in leanness and obesity. Estimated mean cold-induced energy expenditure adjusted for lean body mass, fat mass, and resting energy expenditure **(A)**. The error bars indicated 95% confidence intervals. Comparison of cold-induced thermogenesis (CIT) as percent increase from resting energy expenditure between BAT_neg_ and BAT_pos_ individuals stratified by weight status **(B)**. The graphs indicate median values and interquartile ranges. **p ≤ 0.010.

To better characterize any thermogenic differences between lean and obese participants, we analyzed the effects of cold exposure on energy expenditure, as an indirect marker of BAT thermogenic activity. Cold-induced energy expenditure was adjusted for lean body mass, fat mass and resting energy expenditure to account for the known variation due to differences in body composition. In both weight groups, cold-induced energy expenditure was significantly higher in BAT_pos_ versus BAT_neg_ subjects with no differences between lean and obese participants (**Figure 3A**). The commonly used percent increase in energy expenditure after cold, termed cold-induced thermogenesis (CIT), did not differ between weight groups (**Figure 3B**), suggesting that the functional thermogenic response is similar in leanness and obesity.

### Visceral Obesity and Not Adiposity Per Se Is Negatively Associated With Active BAT

We further investigated the associations between measures of BAT activity and body composition markers in individuals with active BAT (**Table 2**). There was no association between percentage total body fat and BAT activity in any of the study groups. However, the visceral adipose tissue (VAT) volume was negatively correlated with BAT glucose uptake in both lean and obese participants, whereas this association was not found with subcutaneous adipose tissue (SAT) volume. In obese individuals, also BAT volume and CIT correlated negatively with VAT volume which is line with a recent finding of our group (8). In addition, CIT and HOMA-IR were negatively associated in obese participants. Together these data support recent reports that active BAT is associated with improved metabolic parameters particularly in obesity (8, 18).

**Table 2:** Correlation analyses between PET-derived metrics of BAT activity and body composition markers in lean and obese individuals.

	lean				obese			
	%BF	SAT (mL)	VAT (mL)	HOMA-IR	%BF	SAT (mL)	VAT (mL)	HOMA-IR
**BAT volume (mL)**	-0.019	0.046	0.028	0.313	-0.202	-0.135	-0.639**	-0.256
**SUVmean**	0.171	-0.229	-0.500*	0.279	0.020	-0.025	-0.582**	-0.222
**CIT%**	0.071	-0.003	-0.074	0.035	0.224	0.181	-0.281*	-0.375**

%BF, percentage body fat; SAT, subcutaneous adipose tissue; VAT, visceral adipose tissue; HOMA-IR, Homeostatic Model Assessment for Insulin Resistance; BAT, brown adipose tissue; SUVmean, mean standardized uptake value; CIT, cold-induced thermogenesis. Spearman’s rank correlation coefficients display the strength of association between two variables. *P ≤ 0.050, **P ≤0.010.

## Discussion

Despite accumulating evidence that active BAT is associated with improved metabolic health in obesity (8, 16, 18), relatively little is known about potential differences in BAT quantity and quality between leanness and obesity and the metabolic consequences. In our relatively large cohort of 95 lean and obese women and men without any other significant comorbidities, we found that, after a standardized cooling protocol, individuals with obesity have less frequently active BAT (**Figure 1**). In the literature, the frequency of active BAT in lean or mixed populations differs strongly between the studies, which can partly be explained by different cooling and imaging protocols as well as differing age and sex distributions (7, 19, 20). Studies examining only overweight/obese participants reported generally lower percentages of active BAT compared to studies using lean cohorts (16, 19, 21–23). One possible explanation for the lower BAT frequency in obese individuals could be dysfunctional adrenergic signaling in adipose tissue as previously reported in various obesity models (24, 25). In a preclinical study, adipocytes from SAT of obese individuals were less susceptible to norepinephrine-induced browning than adipocytes form lean donors (26). Accordingly, treatment with the sympathomimetic ephedrine increased BAT ^18^F-FDG uptake only in lean but not obese individuals (27). While there are no studies comparing the molecular characteristics of BAT between lean and obese individuals, obesity was associated with the reduced expression of thermogenic genes and beta-adrenergic receptors in both, subcutaneous and visceral WAT (28).

Despite some evidence for a dysfunctional thermogenic response in obesity, recent studies have challenged the view that adiposity or percent body fat content per se are the determining factors for the presence or activity of human BAT (8, 29). Our group found that visceral obesity but not whole-body adiposity is negatively associated with BAT activity (8) while another study even reported a positive association between BAT volume and BMI or percent body fat in men (29). We show here, that despite a lower prevalence of active BAT in obese individuals, BAT volume and SUVmean are similar in lean and obese participants with active BAT (**Figures 1G, H**). Especially unaltered ^18^F-FDG uptake may be unexpected given the general differences in insulin resistance between leanness and obesity. However, cold-induced BAT glucose uptake does not necessarily correlate with glucose uptake in other tissues (30) and we recently reported that active BAT is linked to a healthier metabolic phenotype in obesity including improved insulin resistance and lower VAT mass. VAT is recognized as a source of chronic inflammation associated with the metabolic syndrome which might contribute to “whitening” and dysfunction of BAT (31). Here we found that VAT volume but not percent body fat or SAT volume were inversely associated with BAT activity in lean and obese participants (**Table 2**). In participants with obesity, VAT also correlated negatively with BAT volume and CIT (**Table 2**).

Adult human BAT has been described as a heterogeneous tissue composed of a mixture of brown and white adipocytes (32). Environmental as well as nutritional factors are believed to induce so-called BAT whitening which describes the conversion from multilocular to unilocular non-thermogenic adipocytes which might, in part, be the reason for the regression or absence of BAT activity seen in older or obese individuals (33). Thus, reduced BAT ^18^F-FDG uptake could rather reflect the cellular content, i.e. the ratio between brown and white adipocytes, than the thermogenic potential of individual brown adipocytes in human BAT depots. The almost identical increase in cold-induced energy expenditure and CIT in lean and obese participants with active BAT (**Figure 3**) once again emphasize that the thermogenic capacity of individuals with obesity is intact when BAT is present. At the same time, we recognize that ^18^F-FDG uptake is no direct measure of BAT oxidative metabolism and other tracers such as ^13^C-acetate or fatty acid tracers might be more suitable for this (34). This is because oxidative metabolism of intracellular triglycerides has been shown to be the primary energy substrate for BAT thermogenesis while intermediates of the glycolytic pathway both contribute to thermogenesis and *de-novo* fatty acid synthesis at the same time (35). Our current observations also suggest that the lower abundance of active BAT in obesity might not be driven by overnutrition per se but the inflammatory phenotype associated with visceral obesity.

A limitation of the study is inherent to the ^18^F-FDG-PET/CT technology, widely accepted as the gold standard for BAT detection and quantification. However, as cited several times before (36, 37) we cannot exclude differences in the sensitivity to ^18^F-FDG between lean and obese BAT although similar ^18^F-FDG mean standard uptake values in both weight groups with active BAT do not suggest such a bias.

In summary, we demonstrate here that BAT is less frequently detected in lean versus obese individuals which is not associated with total body fat content per se but with visceral adiposity. When BAT is present, BAT volume, metabolic activity and cold-induced thermogenesis are similar in both weight groups suggesting that BAT can be functional in the obese state. These findings support current and future efforts to harness BAT activity and/or to recruit new BAT as a therapeutic strategy to counteract obesity and its metabolic complications.

## Data Availability Statement

The data sets generated and/or analyzed during this study are available from the corresponding author on reasonable request.

## Ethics Statement

All procedures in this study were in accordance with the ethical standards of the institutional ethics committee of the Medical University of Vienna and with the 1964 Helsinki declaration and its later amendments or comparable ethical standards. All participants provided written informed consent to participate in this study.

## Author Contributions

Study conception and design: FWK, OCK, and CTH. Data collection: CS, CTH, FBL, GP, OCK, MP, and RM. Analysis and interpretation of results: OCK, CTH, ARH, CS, FWK, MP, RM, MH and AKW. Draft manuscript preparation: OCK and CTH. All authors reviewed the results and approved the final version of the manuscript. FWK is the guarantor of this work and, as such, had full access to all the data in the study and takes responsibility for the integrity of the data and the accuracy of the data analysis.

## Funding

This work was supported by the Austrian Science Fund, P 27391 and the Medical Scientific Fund of the Mayor of the City of Vienna 17094, both to FWK.

## Conflict of Interest

The authors declare that the research was conducted in the absence of any commercial or financial relationships that could be construed as a potential conflict of interest.

## Publisher’s Note

All claims expressed in this article are solely those of the authors and do not necessarily represent those of their affiliated organizations, or those of the publisher, the editors and the reviewers. Any product that may be evaluated in this article, or claim that may be made by its manufacturer, is not guaranteed or endorsed by the publisher.
